# Novel High‐Efficient Method to Generate Fragmented Nano‐ and Microfibers Enabling an Additive for Bio‐Inks

**DOI:** 10.1002/mabi.202500251

**Published:** 2025-08-06

**Authors:** Margitta Büchner, Michael Geske, Michael Redel, Dirk W. Schubert

**Affiliations:** ^1^ Institute of Polymer Materials Friedrich‐Alexander‐Universität Erlangen‐Nürnberg Erlangen Germany

**Keywords:** electrospinning, fiber fragmentation, gelatin, polycaprolactone, UV light, Weibull distribution

## Abstract

As an emerging technology, biofabrication combines biopolymers and living cells to create functional tissues, allowing the development of structures that closely mimic native tissues. The use of fiber‐reinforced materials is of particular interest, as it enhances both mechanical properties and cellular behavior. Incorporating fiber fragments into bio‐inks not only strengthens printed structures but also supports cell survival by lowering polymer concentrations and thus the stress exerted on the cells during printing. A key factor in optimizing fiber‐reinforced bio‐inks is the controlled fiber shortening, comprising cutting or breaking, which improves printability and mechanical integrity of printed constructs. However, current methods for fiber fragmentation face significant limitations, including material‐specific dependencies, scalability challenges, and requirements of specialized equipment, which may not be accessible in all laboratories. To overcome these challenges, we introduce a novel approach utilizing ultraviolet irradiation to achieve controlled fiber fragmentation. The average fiber length resulting from specific irradiation times can be estimated using a multi‐modal Weibull analysis. This technique is validated on fibers made of polycaprolactone (PCL) and gelatin blends, demonstrating its cost‐effectiveness, biocompatibility, and simplicity. This study provides a practical solution for fiber fragment production and average length estimation, offering an accessible and scalable alternative for fiber‐based biofabrication applications.

## Graphical abstract

Electrospun fibers are randomly spun on a substrate. Subsequent UV irradiation results in a deliberate breakage of the fibers. The collection of the fragments enables a simple way to make tailor‐made additives for bio‐inks. Dependent on the duration of irradiation, the fiber fragment length distribution changes. The cumulative fiber length distribution is analyzed, yielding fundamental equations for application.

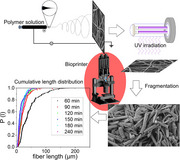

## 1 Introduction

Biofabrication represents a promising technology for the creation of functional tissues and customized implants [1, 2]. By combining printable biopolymers with living cells, it becomes possible to create structures that closely resemble native tissues in both mechanical and biological aspects [3]. Several biofabrication methods exist, as outlined and discussed by Ng et al. in their review paper, including inkjet‐based, vat photopolymerization, or extrusion‐based bioprinting. However, the present study focuses only on the latter, as according to Ng et al., it is the most widely applied method for fabricating 3D tissue models, accounting for about 88% [2]. In this context, fiber‐reinforced materials are of particular interest as they not only influence cellular behavior through their surface topography, stiffness, and degradation dynamics but also enable targeted enhancement of mechanical properties [4]. For instance, Sonnleitner et al. demonstrated that the incorporation of fiber fragments into bio‐inks is crucial for enhancing the mechanical stability and strength of printed structures. At the same time, reducing the polymer concentration not only improves printability but also promotes cell survival. A lower polymer concentration reduces the mechanical stress on cells during the printing process, leading to higher cell viability while maintaining the structural integrity of the printed tissues [5].

There are various methods of fiber production for biological applications, as highlighted by Walsh et al., with electrospinning being a well‐established technique for generating nano‐ and microfibers [6].

For extrusion‐based bioprinting applications, however, it is essential to shorten these fibers, since longer or continuous fibers tend to aggregate or clog the printing nozzle [5, 7]. Lamberger et al. have discussed several approaches for achieving fiber shortening, including both in situ and post‐spinning techniques. Their main conclusion is that the existing fragmentation methods are constrained by factors such as material specifications, limited scalability, or inadequate fragment size control. To address these challenges, they introduced a novel technique for the fragmentation of electrospun fibers using cryo‐sectioning. This method involves the use of a sacrificial membrane as a substrate, which enables precise cutting of the fibers [7]. Although this technique provides precise control over fiber lengths, it also has some limitations. The use of a sacrificial membrane and the requirement of precise cryo‐sectioning may require specialized and dedicated equipment and setup. This could be a challenge for laboratories without the necessary technology or expertise, which limits the broad application of the method. Moreover, for large‐scale production, multiple setups or more advanced equipment might be needed to handle the increased throughput. This could lead to higher initial costs and operational difficulties that may not be feasible for all research institutions or companies.

To overcome this limitation, we introduce an alternative method for the controlled fragmentation of electrospun fibers based on UV irradiation. To our knowledge, this method has not been systematically studied yet within the context of fiber‐based biofabrication. It offers a cost‐effective, accessible, and reproducible alternative that significantly reduces experimental complexity. This approach utilizes deliberate photochemical degradation to shorten the fibers, allowing an estimation of fiber length distribution and e.g., its average and standard deviation, after specific irradiation times by a model description based on a multi‐modal Weibull analysis. We validated the method by applying irradiation times ranging from 60 to 240 min, using a model system comprising polycaprolactone (PCL) and gelatin, a biocompatible polymer blend widely used in biological applications due to its good biodegradability and cell compatibility [8, 9, 10].

Our aim is to provide a practical and efficient tool for both the production of fiber fragments as well as for the estimation of the average fiber length.

## 2 Results and Discussion

### 2.1 Fiber Diameter Distribution

**FIGURE 1:**
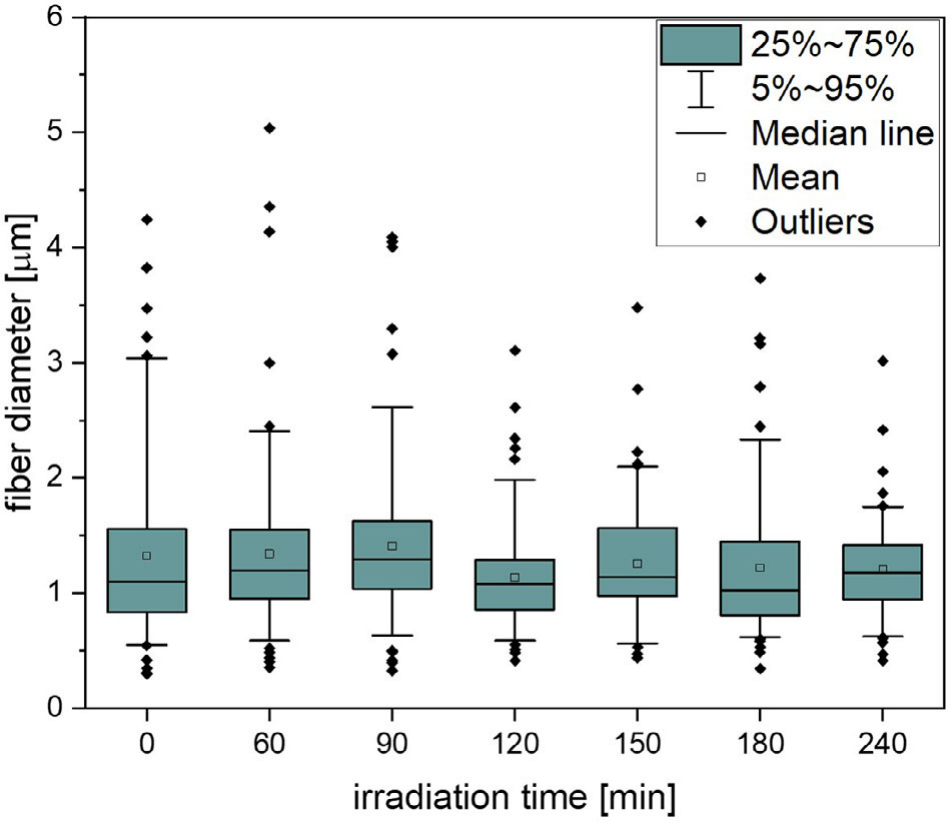
Whisker box plots show the distribution of fiber diameter for non‐irradiated fibers and fiber fragments after an irradiation time of 60, 90, 120, 150, 180, and 240 min. Statistical analysis using a one‐way ANOVA (*p* ≤ 0.01) indicated no significant differences in fiber diameter between the non‐irradiated sample and samples exposed to UV light for various time intervals.

First, the fiber diameter distribution is considered. Figure 1 presents the whisker box plots demonstrating the distribution of the fiber diameter for irradiation times ranging from 0 to 240 min.

The statistical analysis revealed no significant dependence of fiber diameter on the irradiation times. Thus, we assume that the fiber diameter remains constant within the experimental error. Our primary focus is therefore on the fiber fragmentation and the distribution of fiber lengths.

### 2.2 Fiber Length Distribution

**FIGURE 2:**
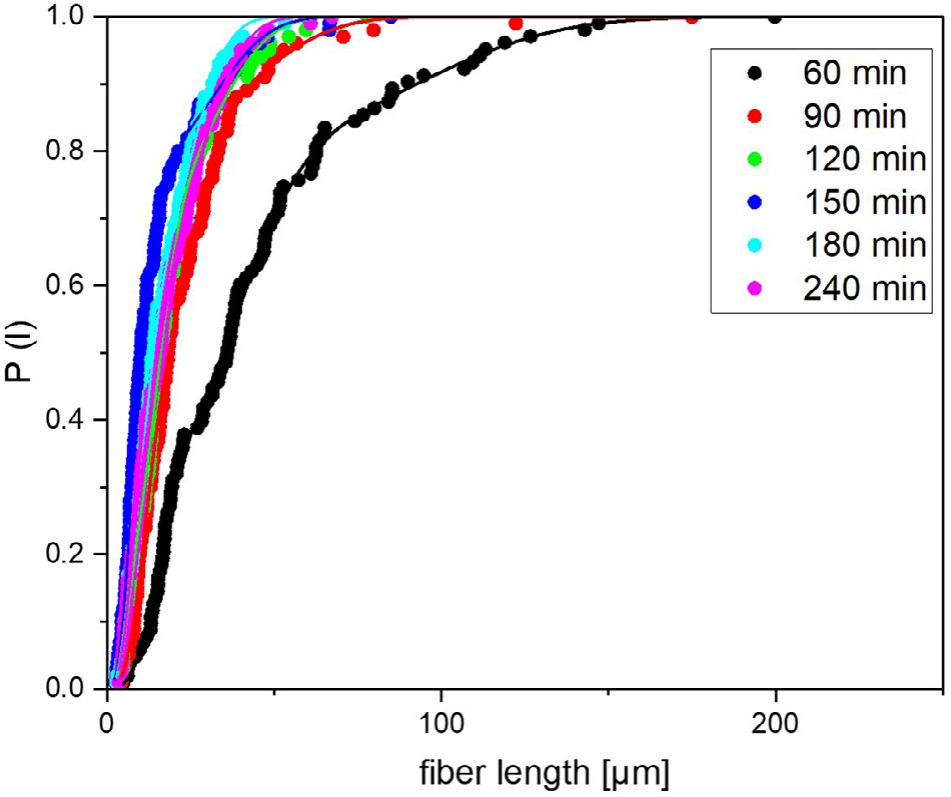
Cumulative experimental fiber length distribution for irradiation times of 60, 90, 120, 150, 180, and 240 min. Dots are the experimental data and the solid line fit curves according Equation (1).

Figure 2 shows the cumulative experimental fiber length distributions, which are fitted by multi‐modal Weibull distributions as described in Equation 1.

(1) $$\begin{array}{cc} & P\;\left(l\right)\\ & =A_{1}\;\left(1-\exp\left(-{\left(\frac{l}{a_{1}}\right)}^{3}\right)\right)+A_{2}\left(1-\exp\left(-{\left(\frac{l}{a_{2}}\right)}^{3}\right)\right)\\ & +A_{3}\left(1-\exp\left(-{\left(\frac{l}{a_{3}}\right)}^{3}\right)\right) \end{array}$$

with

(2) $$\sum_{i=1}^{3}\;A_{i}=1$$

Such an approach is already suggested in the literature [11]. Furthermore, it was successfully applied to describe fiber diameter distribution modes in electrospinning, where the stretch exponent can be set to 3, achieving excellent fits, as evident from Figure 2 [12].

Thus, five freely adjustable parameters remain, where the *a_i_* are called mode lengths in the following text. Table 1 summarizes the achieved fit parameters:

**TABLE 1:** Results from fitting the data according to Equation (1), as shown in Figure 3. Errors for *a_1_* are typically 1 µm, *a_2_* typically 2 µm, *a_3_* typically 4 µm, respectively, while the errors for *A_i_* are typically 20 %.

Irradiation time (min)	a_1_ (µm)	A_1_ (‐)	a_2_ (µm)	A_2_ (‐)	a_3_ (µm)	A_3_ (‐)
60	17	0.31	44	0.48	101	0.21
90	12	0.38	27	0.46	52	0.16
120	7	0.15	17	0.55	37	0.30
150	8	0.46	15	0.31	37	0.23
180	5	0.09	12	0.44	27	0.47
240	8	0.24	15	0.34	31	0.42

**FIGURE 3:**
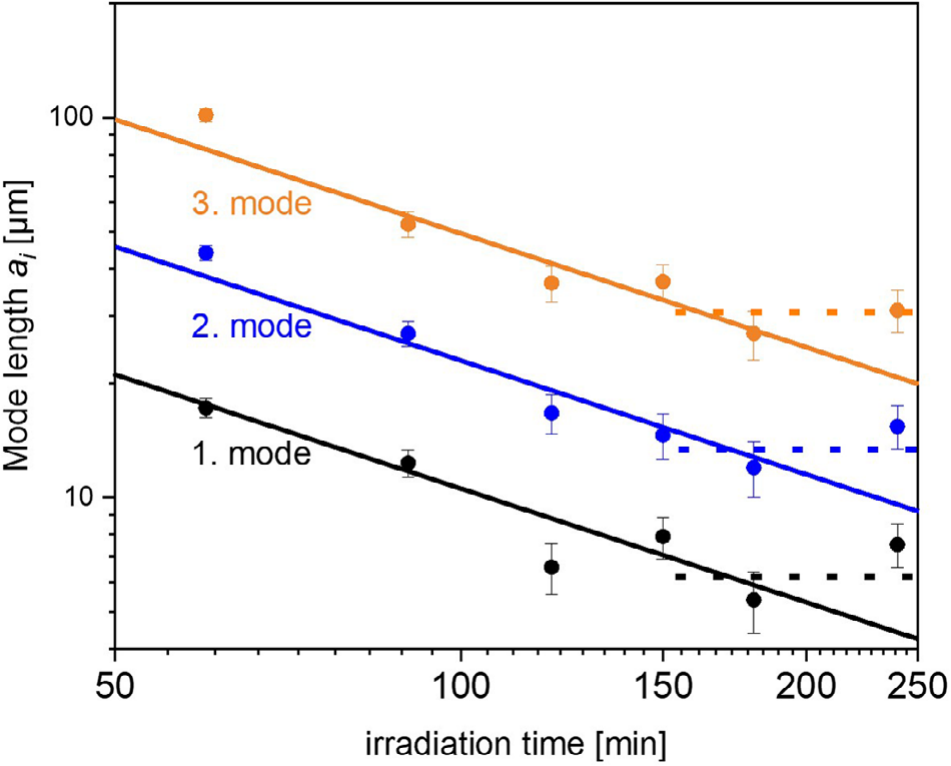
Double log‐plot, mode lengths as a function of irradiation time. Solid lines represent mode length ∼ 1/irradiation time. The dashed lines indicate a potential equilibrium around 150–180 min.

From Figure 3, it is evident that the modes are separated by a factor 2, meaning

(3) $$a_{2}=2\cdot a_{1}$$

(4) $$a_{3}=2\cdot \;a_{2}=4\cdot a_{1}$$

with the mode length being reciprocally proportional to the irradiation time, at least in the investigated experimental range up to approximately 150 min. Above an irradiation time of 150 min, the distribution no longer seems to change significantly.

Nevertheless, these findings will be investigated and challenged in a separate work on the base of computer simulation, where models for the probability of fiber breakage as a function of length must be considered and convoluted with the length distribution after each time step.

In the present work, we thus focus only on reasonably useful experimental results worth communicating to the community utilizing fiber fragments for fiber‐reinforced bio‐inks, seeking methods with higher fiber yield.

**FIGURE 4:**
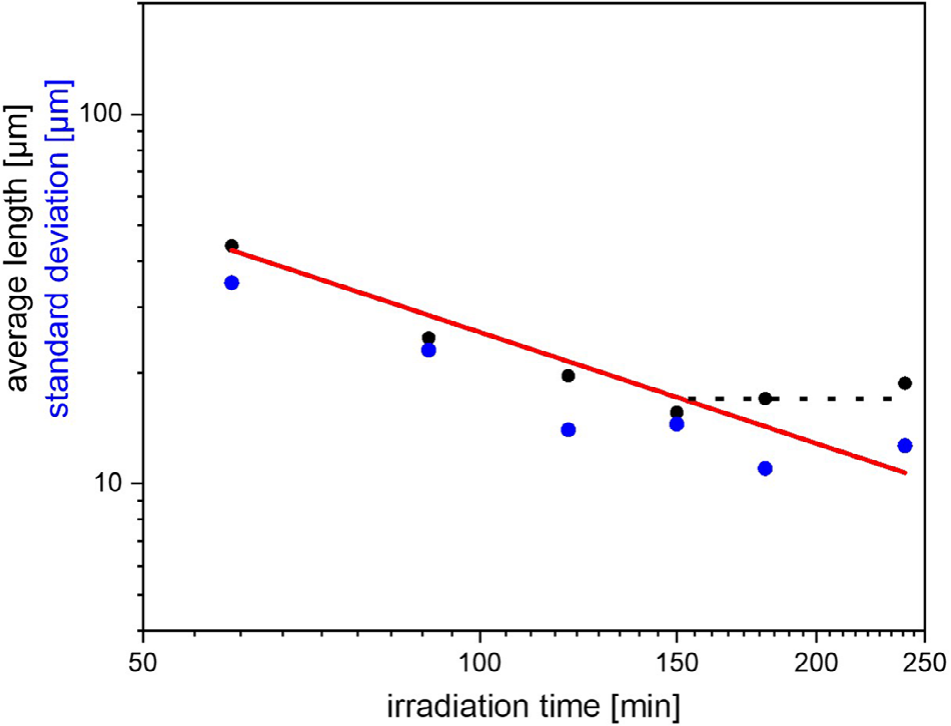
Average length and corresponding standard variation as a function of irradiation time. The solid red line indicates a reciprocal proportionality to irradiation time.

For practical purposes, the average in fragment length and its corresponding standard deviation are shown in Figure 4 as a function of irradiation time.

The rather large potential errors in the A_i_ values and their apparently arbitrary variations result in a trial to enforce A_i_ fits independent on the irradiation time. Such a trial, considering only the data sets for 60, 90, and 120 min irradiation time, reveals that the A_i_ can be set to 1/3, yielding finally:

(5) $$\begin{array}{cc} P\;\left(l\right) & =\frac{1}{3}\;\left(1-\exp\left(-{\left(\frac{l}{a}\right)}^{3}\right)\right)+\frac{1}{3}\left(1-\exp\left(-{\left(\frac{l}{2a}\right)}^{3}\right)\right)\\ & +\frac{1}{3}\left(1-\exp\left(-{\left(\frac{l}{4a}\right)}^{3}\right)\right) \end{array}$$

with

(6) $$a=\frac{1126\;\mu m}{\mathrm{irradtiation}\;\mathrm{time}/\mathrm{minutes}}\;$$

for the range 60–120 min.

The above cumulative distribution (Equation 5) approximately yields the average length

(7) $$\langle l\rangle \;\approx \sqrt{\frac{13}{3}}a$$

And for the standard deviation

(8) $$\sigma \approx \sqrt{2}a$$

Thus, the above Equations (6), (7), and (8) provide useful results for applications toward a tailor‐made effective fiber fragment processing and subsequent utilization in bio‐inks.

The last issue regarding application is the question if the UV irradiation changes the polymer.

Therefore, we applied Fourier‐transform infrared (FTIR) to answer the question.

### 2.3 Fourier‐Transform Infrared (FTIR) Analysis

**FIGURE 5:**
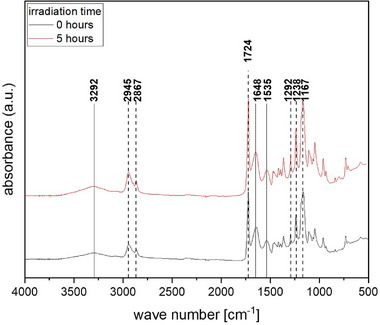
FTIR spectra of PCL/gelatin blend fibers before (black) and after 5 h of UV irradiation (red). The significant peaks of PCL and gelatin demonstrate the presence of both components in the fibers: PCL‐specific peaks represented with dashed lines and gelatin‐specific peaks symbolized with solid lines. No shift in the position of the peaks is observed after 5 h of UV irradiation.

FTIR spectra were recorded for two fiber samples—one non‐irradiated and one exposed to UV irradiation for 5 h—to assess potential chemical changes induced by UV exposure. As shown in Figure 5, the spectra confirm the presence of both PCL and gelatin in the samples, as evidenced by four characteristic absorption bands for PCL at 2945 cm^−1^ (asymmetric CH_2_ stretching), 2867 cm^−1^ (symmetric CH_2_ stretching), 1724 cm^−1^ (carbonyl stretching), and 1293 cm^−1^ (C–O/C–C stretching in the crystalline phase) [9, 13]. Gelatin‐specific peaks were identified at 3292 cm^−1^ (N–H stretching), 1648 cm^−1^ (amide I), and 1535 cm^−1^ (amide II) [9, 14]. A comparison of the spectra revealed no discernible shifts or changes in the position of these peaks, indicating that no chemical alterations occurred in the fiber composition after 5 h of UV exposure.

### 2.4 Scanning Electron Microscopy (SEM)

**FIGURE 6:**
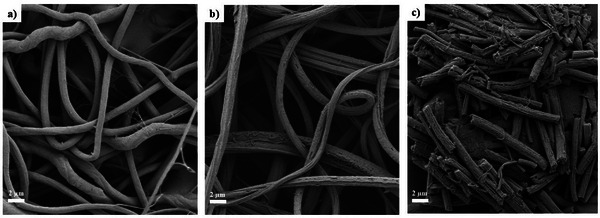
SEM images of (a) non‐irradiated fibers, (b) non‐irradiated fibers after being treated with deionized water, and (c) fiber fragments after 150 min UV light exposure.

Figure 6 displays SEM images of one sample exposed to UV light for 150 min and two non‐irradiated samples, one untreated and one treated with deionized water to ensure comparable conditions to those of the fragmented fibers.

It is remarkable that the non‐irradiated fibers in Figure 6b and the fragmented fibers in Figure 6c exhibit an additional nanostructure compared to the untreated fibers in Figure 6a. This substructure along the fiber direction is attributed to the dispersion of the fibers in water and the subsequent swelling and cracking of the surface. These nanostructured surface features on electrospun fibers can substantially improve cellular responses. For instance, Zamani et al. demonstrated in their study that the presence of nanometer‐scale roughness on porous PLGA fibers significantly promotes the attachment, proliferation, and growth of human nerve cells, which can be explained by the increased surface area available for cell‐material interactions [15]. However, further studies are needed to investigate the interactions between the fiber fragment surface and different cell types. Nevertheless, the nanostructure has a negligible effect on the fiber diameter, as indicated in Figure 1.

### 2.5 Printability Tests

**FIGURE 7:**
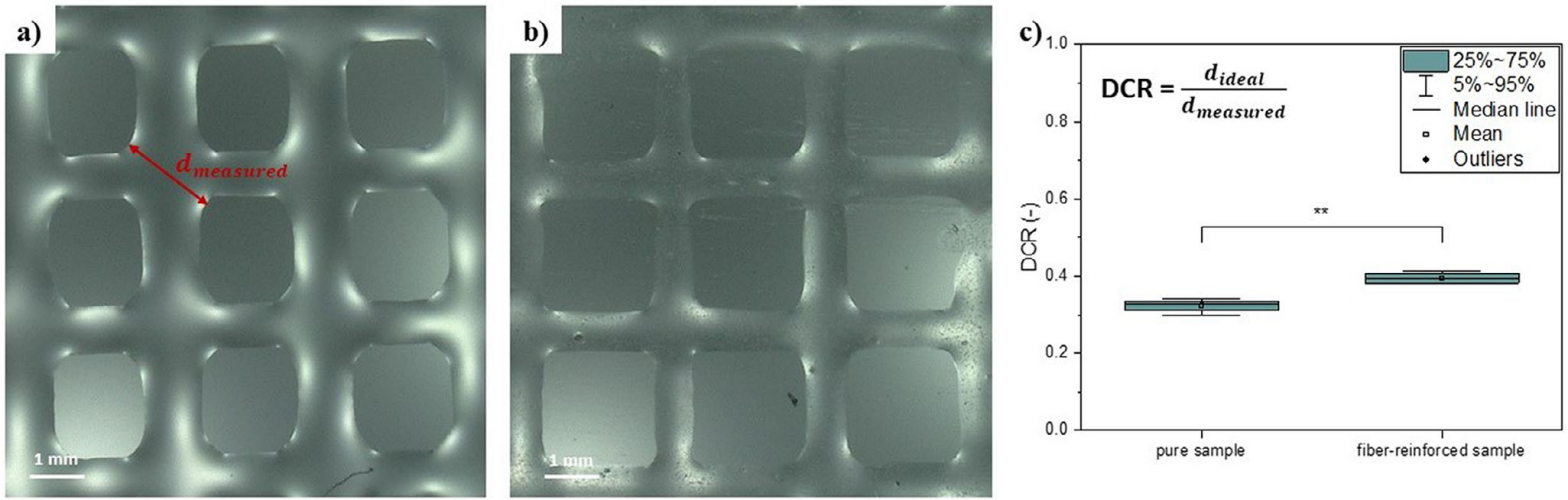
Images of the printed grid structure of 3% (w/v) alginate (a) without fiber fragments and (b) incorporated with 1% (w/v) PCL/gelatin fiber fragments irradiated for 150 min and (c) calculated DCR values of both samples on four intersection points. *p*‐value represents significance level with ^**^ *p* < 0.01.

To demonstrate the proof‐of‐concept, grid structure tests were conducted on 3% (w/v) alginate dissolved in deionized water to assess the effect of incorporating the fragmented fibers on the printability. Two types of samples were compared: one without fiber fragments and one reinforced using 1% (w/v) fiber fragments obtained after 150 min of UV light exposure. Figure 7 shows the images of the printed grid structure for both samples. These structures were evaluated by calculating the diagonal crossing ratio (DCR) which is the quotient of the ideal diagonal *d* (354 µm for a G25 nozzle) to the measured diagonal of the intersection, as illustrated in Figure 7. Accordingly, a value of 1 denotes an ideal printed shape while values < 1 indicate coalescence of the overlapping strands [16]. For the analysis, only the central region of the images was considered, since this study represents a proof‐of‐concept and the methodology has not yet been fully established, consequently, the peripheral areas exhibit lower quality and were therefore excluded from quantitative evaluation.

Notably, the overlapping strands in the pure sample exhibit greater fusion in comparison to the fiber‐reinforced sample, reflected by a calculated DCR of 0.32 ± 0.02. In contrast, the fiber‐reinforced sample attains a higher calculated DCR of 0.39 ± 0.01, with statistical analysis confirming that this increase is significant, indicating an improvement in shape fidelity attributable to fiber incorporation.

## 3 Conclusion

The UV irradiation method for controlled fragmentation of electrospun fibers offers a promising and accessible alternative to existing techniques. It is cost‐effective, easy to implement, and provides reproducible results, effectively addressing challenges such as scalability and complexity. Validation with a PCL and gelatin model system demonstrates its suitability for biocompatible, biodegradable polymers. A statistical analysis yields no significant changes in fiber diameter for irradiation times up to 240 min. FTIR spectra confirmed the successful incorporation of PCL and gelatin, with no shifts observed between the non‐irradiated fibers and the fibers irradiated for 5 h. This suggests that UV irradiation does not affect either the fiber diameter or the chemical composition within the irradiation time range of 0–240 min. Printability tests revealed that the incorporation of fiber fragments significantly enhanced the shape fidelity of the printed construct, as evidenced by the increased diagonal crossing ratio (DCR) of 0.39 ± 0.01 compared to the DCR of the pure alginate sample of 0.32 ± 0.02. The average fiber length and standard deviation resulting from the UV irradiation method can be estimated using an equation for a given irradiation time, providing a valuable tool for practical applications.

## 4 Experimental Section

### 4.1 Materials

PCL (M_W_ = 80 000 g mol^−1^) and gelatin (type A, porcine skin, gel strength 300) were both purchased from Sigma Aldrich (Saint Louis, MO, USA). The solvents formic acid (≥ 98%) and acetic acid (100%) were purchased from Carl Roth GmbH + Co. KG (Karlsruhe, Germany). Alginate PH176 was provided by Vivapharm (JRS Pharma GmbH & Co. KG, Rosenberg, Germany).

### 4.2 Fiber Production

The PCL and gelatin fibers were produced by electrospinning. The electrospinning solution was prepared by dissolving 0.08 g mL^−1^ gelatin and 0.16 g mL^−1^ PCL in a 7:3 mixture of formic acid and acetic acid. This approach was based on the results of a previously published paper by Himmler et al. who showed that gelatin could be successfully incorporated into the PCL matrix [9]. The spinning solution was electrospun in a randomly oriented manner onto an aluminum foil for 14 min at a flow rate of 0.2 mL h^−1^, a voltage of 15 kV and a distance of 15 cm between the needle and collector.

### 4.3 Fragmentation

The electrospun fiber mats were exposed to UV light (Coospider CTUV‐25 25W220VVAC, λ = 253.7 nm) with a distance of 10 cm, with exposure times varying from 60 min to 4 h. The fibers were carefully removed from the aluminium foil with a spatula and subsequently dispersed with an Ultra‐Turrax (IKA ULTRA‐TURRAX T18 basic, Germany) in distilled water at a mixing speed of 11 000 rpm for 30 min.

### 4.4 Fiber characterization

Fiber diameter distributions were analyzed by scanning electron microscopy SEM (CrossBeam Carl Zeiss Microscopy GmbH, Oberkochen, Germany). A drop of the dispersion was placed on a sample holder and left to dry. The specimens were gold‐coated with a Q150T turbo‐pumped sputter coater (Quorum Technologies Inc., Guelph, ON, CA) before being placed into the SEM's vacuum chamber. Fiber length distributions were measured using an optical microscope (VHX7000, Objektiv VH250W, Keyence Deutschland GmbH, Neu‐Isenburg, Germany). The dispersion was inserted into a petri dish to facilitate water evaporation. For every setting, 10 images were taken and 10 fiber diameters or fiber lengths were evaluated in each image using ImageJ/Fiji 1.54f, respectively [17]. FTIR spectroscopy was conducted in attenuated total reflectance (ATR) using a Nicolet iS50 FTIR spectrometer (Thermo Fisher Scientific, Waltham, MA, USA) with a diamond single bounce crystal (refraction angle: 42°), covering a wavenumber range of 4000–500 cm^−1^;. The spectra were recorded for both a non‐irradiated sample and a sample irradiated for 5 h. The measurements were averages of 32 scans at a resolution of 4.0 cm^−1^.

### 4.5 Printability Tests

For analyzing the printability, grid structure tests (GST), developed as a part of the research project SFB/TRR 225 “Biofabrication” (DFG, Deutsche Forschungsgemeinschaft—project number 326998133), were performed. The two‐layer grid structure was generated using a DIY direct drive extrusion setup (used in the research project SFB/TRR 225), equipped with a G25 nozzle with an inner diameter of 250 µm (7018333, Nordson Corporation, Westlake, OH, USA). The resulting structure was printed on a microscope slide (Epredia, Kalamazoo, MI, USA) with a printing velocity of 5 mm s^−1^.

The images were captured 60 s after the printing was completed via an optical microscope (VHX7000, Objektiv VH‐Z20R, Keyence Deutschland GmbH, Neu‐Isenburg, Germany). In each image, the four diagonals of the intersecting strands were subsequently measured using the software ImageJ/Fiji 1.54f [17].

### 4.6 Bioink Preparation

The 3% (w/v) alginate solution was prepared by dissolving the weighed polymer in deionized water with constant stirring. For the fiber‐reinforced sample, the weighed polymer was dissolved in a 1% (w/v) dispersion of fiber fragments in deionized water.

### 4.7 Statistical Analysis

Statistical analyses were conducted using a one‐way analysis of variance (ANOVA) with OriginLab software (Origin, Version 2022. OriginLab Corporation, Northampton, MA, USA). A *p*‐value of less than 0.01 was considered statistically significant.

## Conflicts of Interest

The authors declare no conflicts of interest.

## Data Availability

The data that support the findings of this study are available from the corresponding author upon reasonable request.
